# Supplementary figures and images for: Analyzing Single Molecule Localization Microscopy Data Using Cubic Splines
Source: Sci Rep. 2017 Apr 3;7:552. doi: 10.1038/s41598-017-00622-w (PMC5428856; doi:10.1038/s41598-017-00622-w)

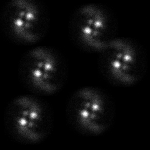

Supplement: Supplementary file 1 — Python code and Data [file 41598_2017_622_MOESM1_ESM.zip › supplement/figure5/sequence-as-stack-Beads-DH-Exp.tif]
